# The relationship between bacterial sources and genotype to the antimicrobial resistance pattern of *Burkholderia pseudomallei*

**DOI:** 10.14202/vetworld.2018.1404-1408

**Published:** 2018-10-12

**Authors:** Muhammad Abubakar Sadiq, Latiffah Hassan, Saleha Abdul Aziz, Zunita Zakaria, Hassan Ismail Musa, Maswati Mat Amin

**Affiliations:** 1 Department of Microbiology and Pathology, Faculty of Veterinary Medicine, Universiti Putra Malaysia (UPM), 44300 UPM Serdang, Selangor Darul Ehsan Malaysia; 2 Department of Veterinary Public Health and Preventive Medicine, Faculty of Veterinary Medicine, University of Maiduguri, P.M.B 1069 Maiduguri, Borno State Nigeria; 3 Regional Veterinary Laboratory, Bukit Tengah, Peti Surat 63, 14007 Bukit Mertajam, Seberang Perai Tengah, Pulau Pinang, Malaysia

**Keywords:** animals, antimicrobial, *Burkholderia pseudomallei*, environmental, resistance, sequence types, veterinary isolates

## Abstract

**Background:**

Melioidosis is a fatal emerging infectious disease of both man and animal caused by bacteria *Burkholderia pseudomallei*. Variations were suggested to have existed among the different *B. pseudomallei* clinical strains/genotypes which may implicate bacterial susceptibility and resistance toward antibiotics.

**Aim:**

This study was designed to determine whether the phenotypic antibiotic resistance pattern of *B. pseudomallei* is associated with the source of isolates and the genotype.

**Materials and Methods:**

A collection of 111 *B. pseudomallei* isolates from veterinary cases of melioidosis and the environments (soil and water) were obtained from stock cultures of previous studies and were phylogenetically characterized by multilocus sequence typing (ST). The susceptibility to five antibiotics, namely meropenem (MEM), imipenem, ceftazidime (CAZ), cotrimoxazole (SXT), and co-amoxiclav (AMC), recommended in both acute and eradication phases of melioidosis treatment were tested using minimum inhibitory concentration antibiotics susceptibility test.

**Results:**

Majority of isolates were susceptible to all antibiotics tested while few resistant strains to MEM, SXT, CAZ, and AMC were observed. Statistically significant association was found between resistance to MEM and the veterinary clinical isolates (p<0.05). The likelihood of resistance to MEM was significantly higher among the novel ST 1130 isolates found in veterinary cases as compared to others.

**Conclusion:**

The resistance to MEM and SXT appeared to be higher among veterinary isolates, and the novel ST 1130 was more likely to be resistant to MEM as compared to others.

## Introduction

Melioidosis caused by the Gram-negative bacteria *Burkholderia pseudomallei* is considered as an emerging infectious disease of both man and animals in highly endemic regions of Southeast Asia and Northern Australia [1]. Even though the treatment is rather challenging following infection, antibiotic chemotherapy has been reported to control the disease and improve patient survival [2]. However, antibiotic treatment of clinical melioidosis is protracted, expensive and often unsuccessful if not properly implemented [3]. For infections in animal in non-endemic areas, prompt isolation of all infected animals and culling is recommended due to the chronic nature of melioidosis [4]. When treatment of animal becomes necessary due to emotional and sentimental values, the treatment regimen in animals follows that of humans; along with animal confinement and biosecurity measures to avoid spread of the agent.

No differences have been observed on the resistance pattern among the *B. pseudomallei* strains obtained from different sources [5]. However, others have suggested that variation exists among the different *B. pseudomallei* clinical strains/genotypes which may implicate bacterial susceptibility and resistance toward antibiotics [6,7]. These variations can potentially be of clinical importance when deciding treatment options following infection. Since melioidosis is a challenging disease to treat, the knowledge about the differences in resistance behavior if they exist, between strains and/or genotypes can be useful.

In Malaysia, increasing number of melioidosis cases has been observed in both animals and humans [8,9]. While many studies have been carried out on the human clinical isolates, little has been done on *B. pseudomallei* from veterinary cases. The variations of antibiotic resistance pattern across the bacterial strains/genotypes found among veterinary isolates and relatedness of the genotype to the resistance pattern observed have not been elucidated in Malaysia or elsewhere.

This study aimed to determine if the phenotypic antibiotic resistance pattern is associated with the genotype of *B. pseudomallei* and their sources. This study describes the relationship between the molecular sequence types (STs) of *B. pseudomallei* isolated from veterinary cases and the environment (soil and water) in Malaysia and the pattern of antimicrobial resistance observed.

## Materials and Methods

### Ethical approval

Ethical approval is not required to pursue this type of study.

### Source of isolates

A collection of 111 *B. pseudomallei* isolates stored at −20°C in 20% glycerol brain heart infusion broth were obtained; (i) environmental isolates from the study by Musa *et al*. [10] and veterinary isolates from the bacteriology laboratory of the faculty of veterinary medicine, Universiti Putra Malaysia (UPM) and (ii) veterinary isolates from the Regional Veterinary Laboratory Bukit Tengah, Pulau Pinang, Malaysia.

All isolates were resuscitated by streaking the thawed stock culture on Trypticase Soy Agar (Oxoid Ltd., Basingstoke, United Kingdom) with 5% horse blood and incubated for 48 h at 37°C. All laboratory analyses were conducted in the Skim Akreditasi Makmal Malaysia (SAMM) accredited Veterinary Bacteriology Laboratory of the Faculty of Veterinary Medicine, UPM. The handling of all live *B. pseudomallei* was performed at BSL 2 with extra precautions and additional personal protective equipment.

### Nucleic acid extraction and polymerase chain reaction (PCR) confirmation of isolates

The bacterial DNA was extracted using DNA extraction kit Qiagen DNeasy^®^ (Qiagen, Germany) according to manufacturer’s instructions. All isolates were confirmed by PCR amplification of 550 bp gene fragment using *B*. *pseudomallei*-specific 16S rRNA region primers (PPM3 - forward primer) 5’-AATCATTCTGGCTAATACCCG-3’ and (PPM4 - reverse primer) 5’-CGGTTCTCTTTCGAGCTCG-3’ obtained from the work of Brook *et al*. [11]. A PCR (50 µl volume) mixture comprised 25 µL of Top Taq Master Mix^®^ (Qiagen, Germany) (containing 10× TopTaq PCR Buffer, 25 mM MgCl_2_, 400 µM of each dNTP 10 × and Taq DNA Polymerase 5 units/µL), CoralLoad 5 µL 1 × concentrate, forward primer 0.5 µM; reverse primer 0.5 µM, 13 µL PCR-grade DNase-free water, and 0.25 µg DNA template. The PCR protocol consisted of 30 cycles of 1 min at 94°C, 30 s at 54°C, and 2 min at 72°C, with a final extension step of 10 min at 72°C [11]. Gel electrophoresis of the PCR products was done using 1.5% agarose gel in 1.5 times TBE at 80 volts for 1 h and viewed with Gel Viewer (BioRad, USA).

### Antimicrobial susceptibility testing

Minimum inhibitory concentration (MIC) evaluation method was conducted. MIC evaluation antibiotic gradient strips of five principal antibiotics recommended in intensive and eradication phase treatments of melioidosis [12]; meropenem ([MEM], 32-0.002 µg/mL), imipenem ([IPM], 32-0.002 µg/mL), ceftazidime ([CAZ], 256-0.015 µg/mL), amoxicillin-clavulanic acid (co-amoxiclav) 2/1 ([AMC], 0.015-256 µg/mL) MIC evaluator™ were obtained from oxoid and trimethoprim-sulfamethoxazole (cotrimoxazole) 1/19 ([SXT], 0.002-32.0 µg/mL E-test strips™ from bioMerieux. The susceptibility test was conducted according to manufacturers’ instructions. The MIC values were read directly from the MIC evaluator and E-test strip MIC gradient scales, where the line of inhibition intersects the strip. The MIC for SXT was read at 80% inhibition according to the manufacturer’s instruction. The MIC interpretative criteria defined for *B. cepacia* and *Pseudomonas aeruginosa* [5,13] was used. Where specific breakpoints were not available for these organisms, standard interpretative criteria of non-*Enterobacteriaceae* were applied [13]. All isolates categorized as “intermediate” following repetition of the test was considered “resistant” for clinical practicality because the isolate in the latter category has an uncertain therapeutic effect by the concentration of the tested antibiotic.

### Multilocus sequence typing (MLST) of isolates

PCR amplification (50 µl volumes) on chromosomal DNA was carried out using the *B. pseudomallei* seven housekeeping genes according to the method described by MLST (http://pubmlst.org/bpseudomallei/). The DNA fragments were purified using MEGA quick-spin™ (iNtRON Biotechnology, Korea) purification kit. Sequencing was done using Sanger sequencing reactions in both directions using the same primers that were used for the initial PCR amplification. For each gene fragment, the sequences from the isolate were aligned and trimmed to appropriate size for that given gene and queried against MLST allele and ST query (http://pubmlst.org/bpseudomallei/) to determine the allele and ST numbers. Where the allele and/or the ST are novel, it was submitted to the MLST curator for verification and subsequent assignment of new allele and/or ST number. All strains, alleles, and the STs obtained in this study have been deposited and at the MLST website (http://pubmlst.org/bpseudomallei/).

### Statistical analysis

All data obtained were entered into Microsoft Excel for Windows 10 and analyzed using JMP10™ statistical package (SAS Institute Inc., Cary, NC, USA). Fisher’s exact test was used to test for association [14]. Isolates with ST164, ST205, ST271, ST1131, ST1338, ST1339, and ST1367 were merged into a single category known as “other STs” in the test for association as their numbers per ST were very low.

## Results

A collection of 111 *B. pseudomallei* isolates comprising 33 (29.73%) from animals, 56 (50.45%) from soil and 22 (19.81%) from water were analyzed in our study. Isolates were molecularly characterized by MLST into 11 distinct STs, namely ST46, ST51, ST84, ST164, ST205, ST271, ST1130, ST1131, ST1338, ST1339, and ST1367, of which the latter five STs are novel (Table-1). Antibiotic gradient MIC susceptibility test method revealed that all or most isolates were susceptible to the antibiotics tested (Table-1). The highest level of resistance was observed for MEM among the veterinary isolates (Fisher’s exact test=4.761; p=0.048).

**Table-1:** The distribution of antimicrobial susceptibility and resistance by E-test MIC method of 111 *B. pseudomallei* isolates from veterinary cases and the environment (soil and water).

Antimicrobial agent	S (%)			R (%)			95% CI on R					

							Veterinary(n=33)		Environmental (n=78)			
		
	Veterinary (n=33)	Environmental (n=78)		Veterinary (n=33)	Environmental (n=78)				Soil		Water	
				
		Soil	Water		Soil	Water	Lower	Upper	Lower	Upper	Lower	Upper
*MEM	29 (87.9)	55 (98.2)	22 (100)	4 (12.1)	1 (1.8)	0	0.97	23.2	−1.7	5.3	−	−
IPM	33 (100)	56 (100)	22 (100)	0	0	0	−	−	−	−	−	−
AMC	33 (100)	54 (96.4)	22 (100)	0	2 (3.6)	0	−	−	−1.3	8.4	−	−
SXT	30 (90.9)	53 (94.6)	21 (95.5)	3 (9.1)	3 (5.4)	1 (4.6)	−0.7	18.9	−0.5	11.3	−4.2	13.3
CAZ	33 (100)	54 (96.4)	22 (100)	0	2 (3.6)	0	−	−	−1.3	8.4	−	−

* MEM=Fisher’s exact test=4.761; P=0.048. MEM=Meropenem, IPM=Imipenem, AMC=Co-amoxiclav, SXT=Cotrimoxazole, and CAZ=Ceftazidime, S=Susceptible, R=Resistant, MIC=Minimum inhibitory concentration, *B. pseudomallei=Burkholderia pseudomallei*, CI=Confidence interval

Table-2 shows the distribution of antibiotic susceptibility and resistance pattern by each of the 11 STs recovered in this study. We found that there was an increase in resistance to MEM in the ST1130 category whereby resistance was observed among three of the eight isolates of this STs. Fisher’s exact test has revealed that there was a statistically significant association between STs and MEM resistance (Fisher’s exact test=11.956; p=0.002).

**Table-2:** Distribution of antibiotic resistance and susceptibility E-test MIC by ST of *Burkholderia pseudomallei*.

Sequence type (ST)	Antibiotics (%)									

	^†^MEM		IPM		AMC		SXT		CAZ	
				
	S	R	S	R	S	R	S	R	S	R
ST46 (n=24)	23 (95.8)	1 (4.2)	24 (100)	0	24 (100)	0	24 (100)	0	24 (100)	0
ST51 (n=45)	45 (100)	0	45 (100)	0	44 (97.8)	1 (2.2)	40 (88.9)	5 (11.1)	45 (100)	0
ST84 (n=26)	25 (96.2)	1 (3.8)	26 (100)	0	25 (96.2)	1 (3.8)	25 (96.1)	1 (3.9)	24 (92.3)	2 (7.7)
ST 1130 (n=8)	5 (62.5)	3 (37.5)	8 (100)	0	8 (100)	0	8 (100)	0	8 (100)	0
*Other STs (n=8)	8 (100)	0	8 (100)	0	8 (100)	0	7 (87.5)	1 (12.5)	8 (100)	0
Total	106 (95.5)	5 (4.5)	111 (100)	0	109 (98.2)	2 (1.8)	104 (93.7)	7 (6.3)	109 (98.2)	2 (1.8)

† MEM=Fisher’s exact test=11.956; P=0.002.

* ST164, ST205, ST271, ST1131, ST1338, ST1339, and ST1367 were merged as other STs in the analysis. MEM=Meropenem, AMC=Co-amoxiclav, SXT=Cotrimoxazole, and CAZ=Ceftazidime, S=Susceptible, R=Resistant, ST=Sequence type, S=Susceptible, R=Resistant, MIC=Minimum inhibitory concentration, *B. pseudomallei=Burkholderia pseudomallei*

## Discussion

No vaccine is currently available that prevents melioidosis. Treatment for melioidosis can be challenging as the disease frequently relapse. The relapse could occur due to the sequestration or dormancy of *B. pseudomallei* in tissue macrophages and other sites [15]. The antibiotics recommended for used in the current clinical chemotherapy for acute phase melioidosis are MEM or IPM and CAZ and for eradication phase are SXT or alternatively AMC [2,12]. These antibiotics are also recommended for the treatment of melioidosis in animals [16] when treatment becomes necessary due to emotional attachments, particularly of pet animals.

Resistance to SXT among the veterinary isolates appeared to be higher than among the environmental isolates; however, the difference was not significant. The level of resistance is consistent with the finding of Ahmad *et al*. [17] who reported 10% resistance among *B. pseudomallei* clinical isolates in Malaysia but is in contrast to the lower resistance reported in clinical isolates of Australia (2.5%) [18] and Singapore (6%) [19]. A much lower resistance of 0.33% with an annual range of 0-0.7% among human *B. pseudomallei* isolates in Thailand was reported by Saiprom *et al*. [20] while Dance *et al*. [21] reported low frequencies of 0.80% (5/620) in Laos and 0% (0/149) in Cambodia. The resistance to CAZ in this study was low (1.8%) but was slightly higher than that reported previously in clinical isolates in Malaysia 0.6% (1/81) [17], Thailand 0.1% (4/4225) [22], and Australia 0.6% (1/170) [23].

MEM and IPM are carbapenem drugs often used as the “antibiotics of last resort” as they possess among the broadest spectrum of activity and greatest potency against Gram-positive and Gram-negative bacteria [24]. In our study, we observed a significant level of resistance to MEM among the isolates from veterinary cases (Fisher’s exact test=4.761; p=0.048) (Table-1) as compared to those from the environment. Carbapenem drugs are subjected to the same mechanism of resistance [25]; therefore, a 4.5% resistance for MEM as opposed to 0% for IPM observed in this study is noteworthy (Table-2). The finding for MEM resistance in this study was similar to that reported by Khosravi *et al*. [7] who observed 4.4% resistance among isolates from clinical cases in Malaysia. The same study also reported difference in the level of resistance between IPM (0.6%) and the MEM. The emergences of MEM resistance among IPM susceptible Gram-negative bacteria have been reported for *Klebsiella pneumonia* [26] and *P. aeruginosa* [27] but have not been reported for *B. pseudomallei*. The mechanism of how this occurs is still being studied. Nonetheless, in the study by Pragasam *et al*. [27] on *P. aeruginosa*, it was described that chromosomally mediated mechanisms are specific for each carbapenem due to the target-specific uptake and pumping out of carbapenems based on their structural difference. These mechanisms contribute to different types of resistance phenotypes such as type I (IPM-resistant MEM susceptible - IRMS), type II (MEM-resistant IPM susceptible - MRIS), and type III (IPM-resistant MEM resistant - IRMR) [27]. The finding in our study is similar to “type II” phenotype of resistance. Carbapenem resistance is a significant emerging public health issue and in recent years has resulted in mortalities among patients infected with multiple drug-resistant organisms [28].

No differences of antibiotic resistance between clinical or environmental strains were observed by the studies of Thibault *et al*. [5] and Wuthiekanun *et al*. [22]. However, in our study, MEM and cotrimoxazole resistances of ST1130 and ST51, respectively, were observed in isolates from infected animals and not from environmental isolates. The veterinary isolates of novel ST1130 demonstrated increased resistance to MEM (Fisher’s exact test=11.956; p=0.002 [Table-2]) when compared to other ST. In a previous study, Podin *et al*. [6] described the relationship between antibiotic sensitivity and *B. pseudomallei* phylogeny where a rare sensitivity to gentamicin among clinical isolates of *B. pseudomallei* in Sarawak (Malaysian Borneo) was restricted to genetically related strains that belonged to ST881 or its single-locus variant, ST997. In this study, the novel ST1130 was seen to have increased resistance to MEM; however, it is not clear whether genetic mutation or reassortment that resulted in the emergence of the new ST has any relationship to the increased resistance. We speculated that increased resistance among the veterinary isolates could have occurred because of previous exposures to antibiotics such as β-lactams, through veterinary treatments and/or husbandry practices. The use of antimicrobials in veterinary medicine and the subsequent emergence of resistance such as beta-lactamase, particularly extended-spectrum β-lactamases in animals, are being discussed. However, a significant body of the scientific community supports the link between multidrug-resistant organisms and antimicrobial use in veterinary medicine [29]. More data and further studies are required to confirm our findings, and to ascertain if genetic mutation or reassortment has resulted in the increased resistance.

## Conclusion

Most of the *B. pseudomallei* strains in our study were susceptible to all antibiotics tested. The existence of a few resistant strains suggests a significant threat to the management of infected patients if this resistance trend was to continue. The frequency of resistance to MEM was higher among veterinary isolates compared to the environmental isolates with the novel ST 1130 more likely to be resistant to MEM as compared to others.

## Recommendation

The present study did not include determination of molecular mechanisms of antimicrobial resistance; however, we recommend that there is need to elucidate the mechanisms of resistance to MEM and cotrimoxazole among isolates from animal cases of melioidosis and their relatedness to the isolate phylogeny.

## Authors’ Contributions

MAS, LH, SAA, and ZZ designed the study. MAS conducted the experimental work, while HIM and MMA assisted with stock cultures. MAS and LH drafted the manuscript and corrected it. All authors read and approved the final manuscript.
